# Upcycling 3D Printing PLA Waste into Functional Electrospun Membranes: Effect of Polymer Concentration on Morphology, Surface Properties and Particle Filtration Efficiency

**DOI:** 10.3390/polym18060769

**Published:** 2026-03-22

**Authors:** Manuel J. Torres-Calla, Geraldine Denise Bazan-Panana, Fatimah N. Jacinto, Diego E. Velásquez, J. I. Gonzáles-Coronel, Manuel Chávez-Ruiz, María Verónica Carranza-Oropeza, J. Quispe-Marcatoma, C. V. Landauro

**Affiliations:** 1 Facultad de Ciencias Físicas, Universidad Nacional Mayor de San Marcos, Lima 15081, Peru; manuel.torres@unmsm.edu.pe (M.J.T.-C.); diego.espinoza7@unmsm.edu.pe (D.E.V.); juan.gonzales19@unmsm.edu.pe (J.I.G.-C.); clandauros@unmsm.edu.pe (C.V.L.); 2 Facultad de Química e Ingeniería Química, Universidad Nacional Mayor de San Marcos, Lima 15081, Peru; geraldine.bazan@unmsm.edu.pe (G.D.B.-P.); fatimah.jacinto@unmsm.edu.pe (F.N.J.); mchavezr@ins.gob.pe (M.C.-R.); mcarranzao@unmsm.edu.pe (M.V.C.-O.); 3 Centro de Investigaciones Tecnológicas, Biomédicas y Medioambientales, Bellavista, Callao 07006, Peru

**Keywords:** recycled PLA, electrospinning, fiber morphology, particulate filtration, quality factor

## Abstract

This study investigates the reutilization of polylactic acid (PLA) waste generated by 3D printing through its transformation into electrospun membranes with tunable morphological, surface, thermal, and filtration properties. Polymer solutions containing 5–10 wt % recycled PLA were prepared in a dichloromethane/dimethylformamide system and characterized in terms of viscosity and electrical conductivity. Increasing PLA concentration raised solution viscosity (41.87–339.83 mPa·s) and reduced conductivity (7.63–1.63 µS·cm^−1^), promoting the formation of bead-free fibers with larger diameters (0.221–1.213 µm) and enhanced hydrophobicity (contact angles 112.34–124.38°). FTIR confirmed preservation of the polymer chemical structure after recycling and electrospinning, while DSC revealed reduced crystallinity in the fibrous membranes. Exploratory correlation analysis indicated consistent associations between solution properties, fiber morphology, and wettability. Increasing the number of electrospun layers (1–3) generated denser networks with reduced pore size and improved particle retention. Filtration tests conducted under controlled airflow conditions (85 L min^−1^, 1 cm s^−1^ frontal velocity, 50 cm^2^ effective area) showed removal efficiencies above 90% for PM2.5 and PM5, while PM1 capture improved with increasing membrane thickness. Quality factor analysis highlighted the trade-off between filtration efficiency and pressure drop, identifying intermediate multilayer configurations as providing a favorable balance. These findings demonstrate that electrospinning offers an effective strategy for converting recycled PLA into structurally tunable membranes with adjustable filtration performance, supporting sustainable valorization of additive manufacturing waste.

## 1. Introduction

Environmental pollution, in its various forms, remains one of the most pressing global challenges of the twenty-first century. Among the various contributors, plastic waste has attracted particular attention due to its persistence in natural ecosystems, widespread production, and limited recycling efficiency [1]. The rapid expansion of additive manufacturing technologies, especially fused filament fabrication (FFF), has further contributed to the generation of polymeric waste streams derived from failed prints, support structures, calibration specimens, and discarded prototypes [2,3].

Polylactic acid (PLA) is one of the most widely used thermoplastics in FFF due to its bio-based origin, relatively low processing temperature, ease of printing, and perceived environmental advantages [4]. However, the increasing consumption of PLA in additive manufacturing has led to a growing accumulation of post-processing waste. Furthermore, repeated thermal and mechanical cycles during filament extrusion and printing may induce chain scission, reducing molecular weight and compromising the mechanical performance of recycled PLA, thereby limiting its direct reuse in closed-loop filament production [5,6,7]. In addition, a considerable fraction of the material employed during the design and prototyping stages of additive manufacturing is discarded, generating distributed waste streams that are difficult to recover through conventional recycling routes. For this reason, alternative strategies focused on the upcycling of PLA waste into value-added materials have gained increasing attention as part of circular economy approaches for polymer management [8,9].

Although PLA is commonly described as a biodegradable polymer, its degradation primarily occurs under controlled industrial composting conditions characterized by elevated temperature, humidity, and microbial activity. Such conditions are rarely achieved in natural environments or conventional landfill systems, resulting in significantly slower degradation rates than often assumed [10,11]. Consequently, alternative valorization pathways that transform PLA waste into high-value functional materials have gained increasing attention as part of circular economy strategies for sustainable polymer management [12,13].

Electrospinning has emerged as a versatile and scalable technique for producing continuous polymer fibers with diameters ranging from the micro- to nanometer scale [14,15]. By applying a high-voltage electric field to polymer solutions, electrospinning enables the fabrication of fibrous membranes with high surface area-to-volume ratios, interconnected porosity, and tunable morphology. These structural characteristics make electrospun materials particularly attractive for applications in air filtration, water treatment, biomedical engineering, and controlled drug delivery [16,17]. In the case of PLA, electrospinning has been widely reported as an effective method to produce nanofibrous membranes with high porosity and small pore sizes, which are desirable characteristics for the capture of airborne particulate matter. Several studies have demonstrated that electrospun PLA membranes can achieve high filtration efficiencies while maintaining relatively low pressure drops, highlighting their potential as sustainable alternatives to conventional petroleum-based filtration materials [18,19,20].

Recent studies have demonstrated the feasibility of electrospinning recycled polymers for functional applications. Zander et al. reported the production of nanofibrous membranes from recycled polyethylene terephthalate (PET), expanded polystyrene (EPS), and polycarbonate, achieving filtration efficiencies exceeding 99% for micrometric particles [21]. Similarly, Chinchillas et al. highlighted the potential of electrospun recycled polymers for environmental remediation and advanced textile applications [22]. In the specific case of PLA, several investigations have focused on tailoring fiber morphology through solvent systems, polymer blends, and processing parameters, demonstrating the strong influence of solution viscosity, conductivity, and molecular interactions on fiber formation [23,24,25].

Beyond morphological control, electrospinning also enables the design of functional membranes with tunable interfacial and filtration properties. For instance, Nicosia et al. developed antimicrobial electrospun PLA-based membranes for aerosol filtration, while Eang et al. demonstrated oil-selective absorption using PLA nanofibrous mats, highlighting the versatility of electrospun PLA in environmental applications [26,27].

Despite these advances, further investigation is required to better understand how solution properties and electrospinning parameters influence fiber formation and functional performance when recycled PLA is used as a feedstock. In particular, the relationships between polymer concentration, rheological behavior, electrical properties, fiber morphology, wettability, and filtration efficiency remain insufficiently explored.

In this context, the present study investigates the fabrication of electrospun nanofibrous membranes from recycled PLA derived from 3D printing waste. The influence of polymer concentration on solution properties, fiber morphology, surface wettability, and particle filtration performance is systematically analyzed. The findings contribute to the development of sustainable filtration materials and support the implementation of circular economy strategies for biodegradable polymers in additive manufacturing.

## 2. Materials and Methods

### 2.1. Reagents

Polylactic acid (PLA) scraps generated during the printing of prototypes using a desktop 3D printer (Ultimaker, Utrecht, The Netherlands) were used as the raw material. The filament corresponded to commercial Ultimaker PLA (diameter 2.85 ± 0.10 mm), manufactured from renewable resources and commonly employed for fused deposition modeling (FDM) applications. Prior to processing, the PLA remnants were washed with isopropyl alcohol (≥99.8%, Sigma-Aldrich, Merck KGaA, Darmstadt, Germany) to remove surface contaminants and printing residues, and subsequently dried at 40 °C for 4 h to eliminate residual moisture. PLA dissolution was carried out using an organic solvent mixture composed of dichloromethane (DCM, CDH, New Delhi, India) and dimethylformamide (DMF, Supelco, Bellefonte, PA, USA), both of commercial grade. These solvents were selected based on their proven ability to dissolve aliphatic thermoplastic polymers and their chemical compatibility with PLA, enabling the preparation of homogeneous electrospinning solutions.

### 2.2. Preparation of the Polymer Solution

Polymer solutions were formulated using a solvent mixture of dichloromethane (DCM) and N,N-dimethylformamide (DMF) in a 6:4 volume ratio (DCM:DMF). This composition was chosen for its demonstrated effectiveness in dissolving PLA, facilitating the disentanglement of polymer chain, and ensuring adequate spinnability for electrospinning. Recycled PLA was incorporated into the solvent system to achieve three different concentrations: 5 wt %, 7.5 wt %, and 10 wt %. Each mixture was magnetically stirred in 250 mL borosilicate glass flasks at 300 K and 500 rpm for 24 h to ensure complete dissolution. To minimize solvent evaporation and maintain compositional stability throughout the stirring process, each flask was connected to a water-cooled reflux condenser, regulated at 16 °C using a recirculating thermostatic bath (Julabo F12, Seelbach, Germany).

### 2.3. Characterization of Polymer Solutions

#### 2.3.1. Dynamic Viscosity

The viscosity of the PLA solutions was measured at room temperature using a Biuged BGD 152/1S digital rotational viscometer (Biuged Instruments, Guangzhou, China). Measurements were performed using spindle No. 2 operating at 60 rpm. Each sample was analyzed in triplicate to ensure reproducibility. The results are reported as mean ± standard deviation.

#### 2.3.2. Electrical Conductivity

Electrical conductivity was determined using a HI9033 multi-range conductivity meter (HANNA Instruments, Woonsocket, RI, USA), previously calibrated with a standard solution of 1413 μS·cm^−1^. Measurements were conducted at 25 °C. Each sample was analyzed in triplicate to ensure reproducibility. Conductivity values are expressed in μS·cm^−1^ and reported as mean ± standard deviation.

### 2.4. Electrospinning Process

Electrospinning was carried out utilizing a semi-industrial Innovenso PE-550 electrospinning platform (Innovenso Ltd., Istanbul, Turkey), equipped with a multi-needle configuration consisting of three parallel 16-gauge stainless-steel needles (inner diameter: 1.2 mm). The polymer solution was delivered at a controlled rate of 16 mL·h^−1^ through a precision syringe infusion pump integrated into the system. The applied flow rate was selected to ensure continuous jet formation while preventing bead formation or jet instability, which may occur at excessive feeding rates. A high-voltage potential of 28 kV was applied between the needle tips and the flat metallic collector, providing sufficient electrostatic force to overcome surface tension and promote stable fiber stretching. The tip-to-collector distance was fixed at 160 mm to allow adequate jet flight time for solvent evaporation prior to fiber deposition. All electrospinning procedures were conducted in an environmental chamber where temperature and relative humidity were maintained at 25 ± 1 °C and 50 ± 5%, respectively. These conditions were selected to minimize the influence of ambient fluctuations on solvent evaporation rate, fiber morphology, and process reproducibility.

A series of polymer solutions with concentrations of 5, 6.25, 7.5, 8.75, and 10 wt % was prepared and electrospun under controlled conditions. These concentrations were systematically selected to evaluate their influence on fiber formation, morphology, and overall membrane structure. The purpose of this experimental design was to identify the concentration range that enables the formation of uniform fibers with reduced bead defects and controlled diameter distribution. The designation of the electrospun samples according to PLA concentration is summarized in Table 1.

Based on these observations, the most suitable condition was selected for subsequent experiments involving multilayer membrane fabrication. This approach enabled further tuning of membrane architecture with the aim of improving filtration performance and overall membrane quality.

### 2.5. Characterization of Electrospun Membranes

#### 2.5.1. Fourier-Transform Infrared Spectroscopy (FTIR)

The chemical structure of the PLA-based materials was analyzed using Fourier-transform infrared (FTIR) spectroscopy. Spectral data were acquired using a Bruker ALPHA II spectrometer (Bruker Optik GmbH, Ettlingen, Germany) equipped with an attenuated total reflectance (ATR) module. Measurements were performed within the spectral range of 4000–400 cm^−1^, with a resolution of 4 cm^−1^ over 32 scans.

#### 2.5.2. Differential Scanning Calorimetry (DSC)

The thermal behavior of the electrospun membranes was evaluated using differential scanning calorimetry (DSC). Measurements were performed on a TA Instruments DSC2500 calorimeter (TA Instruments, New Castle, DE, USA) equipped with hermetic Tzero aluminum pans and operated under a nitrogen purge at 50 mL/min. Approximately 2 mg of each sample was sealed in the pans and subjected to an initial equilibration at 20 °C, followed by a heating scan from 20 to 250 °C at a rate of 3 °C/min. The resulting thermograms were analyzed to identify the thermal transitions characteristic of PLA-based materials.

#### 2.5.3. Scanning Electron Microscopy (SEM)

The surface morphology of the electrospun membranes was examined by scanning electron microscopy (SEM) using a Prisma E microscope (Thermo Fisher Scientific, Bleiswijk, The Netherlands) operated at 10 kV. Prior to imaging, membrane samples were sputter-coated with a thin gold layer (approximately 10 nm) using a Quorum Q150R ES rotary-pumped coater (Quorum Technologies, Lewes, UK) to improve conductivity and image quality. Fiber diameters and pore structures were analyzed using ImageJ software version 1.53t, based on SEM micrographs from five randomly selected regions per sample. At least 100 fibers were measured per condition, and data were reported as mean ± standard deviation.

#### 2.5.4. Surface Wettability

The assessment of surface wettability was evaluated through static contact angle measurements performed with a CITBM EMACO-I goniometer (Center for Technological, Biomedical and Environmental Research, Lima, Peru). A 5 μL ultrapure water droplet was deposited on the membrane surface using a calibrated microsyringe, and images were captured 5 s after deposition to calculate the contact angle using ImageJ software version 1.53t. Each sample was measured in triplicate, with results reported as average ± standard deviation.

### 2.6. Correlation Analysis

Statistical correlations among the properties of polymer solutions and the electrospun membranes were evaluated using Pearson’s correlation coefficient, which provides a quantitative measure of the strength and direction of the linear relationship between two continuous variables. The analysis incorporated data obtained from various characterization techniques, including polymer viscosity and conductivity, as well as membrane contact angle and average fiber diameter. The Pearson correlation coefficient was calculated using Equation (1):

(1) $$r_{xy}=\frac{n\sum x_{i}y_{i}-\sum x_{i}\sum y_{i}}{\sqrt{n\sum x_{i}^{2}-(\sum x_{i}^{2})}\sqrt{n\sum y_{i}^{2}-{\left(\sum y_{i}\right)}^{2}}}$$

where *n* is the number of paired observations, and x_i_ and y_i_ represent the corresponding values of the variables under analysis.

This statistical approach enabled the identification of potential linear relationships between the solution properties and the resulting membrane characteristics, contributing to a better understanding of how variations in polymer concentration influence the performance of recycled PLA membranes.

### 2.7. Filtration Efficiency Evaluation

**Figure 1:**
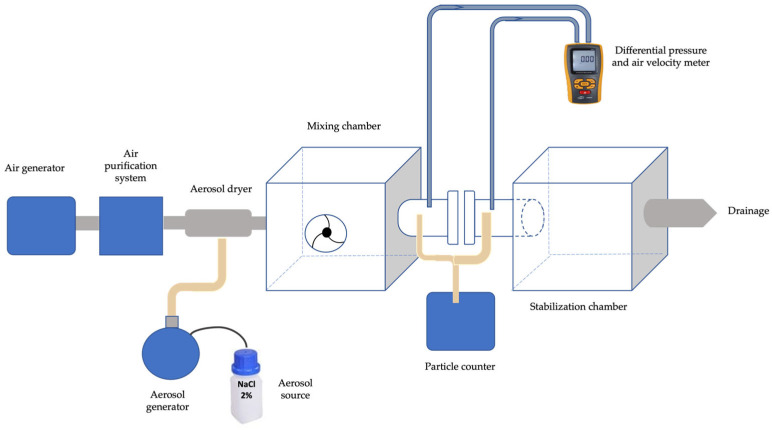
Schematic diagram of the custom-built experimental setup used for particle filtration efficiency (PFE) evaluation. The system includes an air generator and purification unit, a 2 wt % NaCl aerosol generator coupled to an aerosol dryer, a mixing chamber for aerosol homogenization, a membrane holder connected to a laser particle counter, a differential pressure and air velocity meter, and a downstream stabilization chamber prior to drainage.

The particle filtration efficiency (PFE) of the electrospun membranes was evaluated using a custom-built experimental setup inspired by the basic operating principles of commercial Particle Filtration Efficiency (PFE) testers but implemented using lower-cost analogous components. As schematically illustrated in Figure 1, the system consisted of an air generation and purification unit, a 2 wt % NaCl aerosol generator connected to an aerosol dryer, a mixing chamber to homogenize the aerosol stream, a membrane holder coupled to a laser particle counter, a differential pressure and air velocity meter, and a downstream stabilization chamber. The aerosol flow was finally directed to a drainage outlet. All components were integrated into a single experimental platform and operated through a dedicated computer interface [28].

During testing, each membrane sample was mounted in the sample holder and positioned perpendicular to the incoming aerosol flow. The particle counter recorded particle concentrations upstream and downstream of the membrane for three particle size ranges: PM1, PM2.5, and PM5. The particle filtration efficiency was calculated according to Equation (2):

(2) $$\;PFE\left(\%\right)=\;\left[1-\frac{N_{after}}{N_{before}}\right]\times 100\%\;$$

where N_before_ and N_after_ correspond to the upstream and downstream particle counts, respectively.

Tests were conducted on membranes with one, two, and three electrospun layers prepared from the R-PLA10 solution. All measurements were performed at controlled laboratory conditions (25 ± 1 °C, 50 ± 5% RH), and results were expressed as mean ± standard deviation from three independent replicates for each configuration.

### 2.8. Quality Factor

Based on the above considerations, the filtration performance was further evaluated by calculating the quality factor (QF), which integrates filtration efficiency and pressure drop into a single performance parameter. The QF is defined as shown in Equation (3):

(3) $$QF=\;-\;\frac{\ln\left(1-PFE\right)}{\Delta P}$$

This parameter enables the evaluation of the balance between filtration efficiency and air permeability of filtering membranes [29]. A high QF value indicates optimal performance, as the membrane achieves high filtration efficiency with low resistance to airflow, resulting in more comfortable and energy-efficient systems (e.g., face masks with good breathability and high particle retention capacity). In contrast, a low QF reflects that the achieved efficiency relies on a substantial pressure drop, which hinders breathing in personal protective equipment or increases energy consumption in ventilation systems.

## 3. Results and Discussion

### 3.1. Psycochemical Properties of PLA Solutions

Viscosity is defined as the resistance of a fluid to flow under an applied shear stress and is closely related to intermolecular interactions and chain entanglement within polymer solutions. This parameter plays a critical role in electrospinning, as it directly influences jet formation, stability of the Taylor cone, and the resulting fiber morphology [30]. Electrical conductivity, in turn, reflects the ability of a solution to transport electric charges and is a key factor governing jet stretching, fiber diameter reduction, and overall process stability during electrospinning [31].

In the present study, viscosity measurements were performed using a rotational viscometer operating at a fixed spindle speed, providing apparent viscosity values under controlled and reproducible shear conditions. Although polymer solutions such as PLA typically exhibit non-Newtonian behavior, apparent viscosity measured at a constant shear condition is widely used in electrospinning studies as a practical comparative parameter to evaluate concentration-dependent changes in chain entanglement and flow resistance [32]. The use of a fixed shear condition allows consistent comparison among formulations and provides representative values relevant to the shear regime experienced during solution delivery through the electrospinning needle.

Both viscosity and electrical conductivity were measured under identical experimental conditions to assess their combined influence on electrospinning performance. As polymer concentration increased, a marked rise in apparent viscosity was observed, attributable to enhanced chain entanglement and intermolecular interactions within the solution. Conversely, electrical conductivity progressively decreased with increasing PLA content, reflecting the intrinsically low polarity of PLA and the consequent reduction in charge mobility within the solvent system. These trends are consistent with well-established electrospinning theory and previous reports on PLA solutions [32,33].

**Figure 2:**
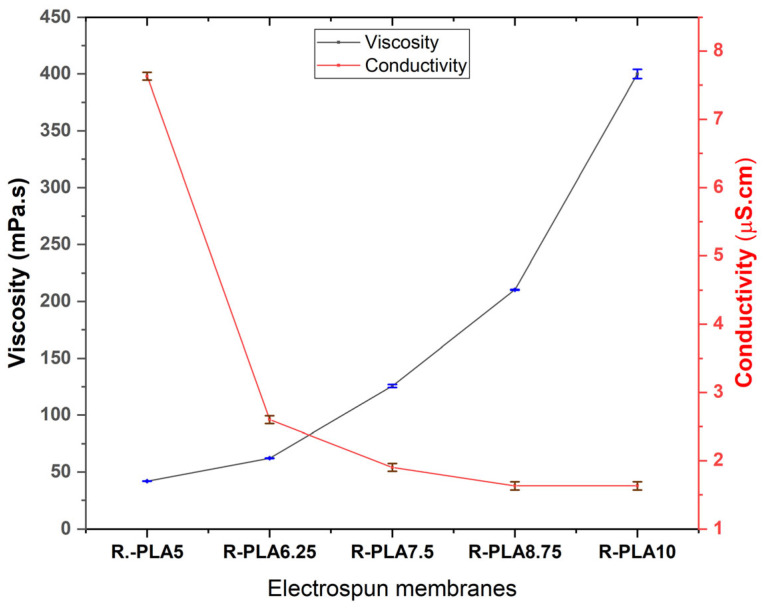
Dynamic viscosity and electrical conductivity of PLA polymer solutions as a function of polymer weight fraction.

As shown in Figure 2, the reduction in electrical conductivity with increasing R-PLA concentration aligns with the behavior reported by Ibili et al. [34], who observed a similar decrease in conductivity in PLA solutions due to the electrically neutral nature of the polymer. At the same time, the continuous increase in viscosity reflects the progressive formation of an entangled polymer network, which is essential for stable fiber formation. Solutions with insufficient viscosity tend to produce bead defects due to inadequate chain entanglement, whereas excessively viscous systems may hinder solution flow and promote needle clogging during electrospinning [16,32,35].

Overall, the results suggest the existence of an optimal concentration window in which a balance between viscosity and electrical conductivity is achieved. This balance is crucial for ensuring jet stability, continuous fiber formation, and uniform membrane morphology, while avoiding defects associated with either insufficient chain entanglement or excessive solution resistance to flow.

### 3.2. Characterization of Electrospun Membranes

#### 3.2.1. Fourier-Transform Infrared Spectroscopy (FTIR)

**Figure 3:**
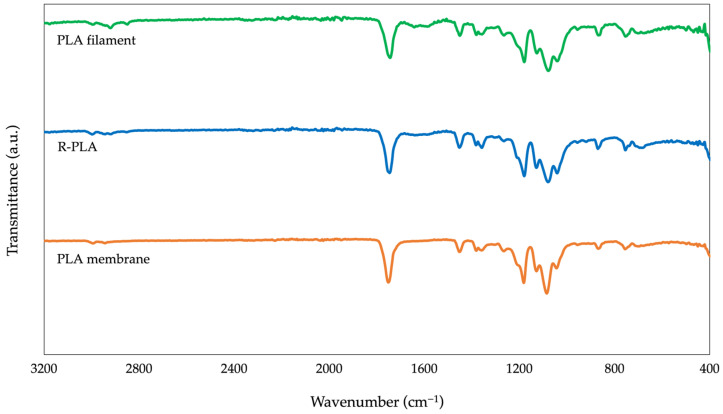
Fourier transform infrared (FTIR) spectra of the pristine PLA filament, recycled PLA (R-PLA) feedstock, and electrospun PLA-based membranes. The spectra were normalized and vertically offset for clarity.

Fourier-transform infrared spectroscopy (FTIR) was employed to compare the chemical structure of the pristine PLA filament, the recycled PLA recovered from 3D-printing waste (R-PLA), and the electrospun PLA membrane. The corresponding spectra are presented in Figure 3.

All materials exhibit the characteristic vibrational bands of polylactic acid, confirming the preservation of the main polyester backbone after both recycling and electrospinning. The intense absorption band observed at approximately 1750 cm^−1^ corresponds to the stretching vibration of the ester carbonyl group (C=O), while the bands located at 2995 and 2945 cm^−1^ are associated with asymmetric and symmetric stretching of methyl groups. Additional peaks at 1450 and 1360 cm^−1^ arise from C–H bending vibrations, and the bands within the 1180–1080 cm^−1^ range correspond to C–O–C stretching modes of the ester linkage, which are characteristic of PLA molecular structure [36,37,38,39,40].

Only minor variations in band intensity and peak sharpness were observed among the three materials. In particular, the electrospun membrane exhibits slightly broader absorption bands, which can be attributed to reduced molecular packing and altered chain conformations induced by solvent dissolution and rapid solidification during electrospinning. Similarly, subtle spectral differences detected in the recycled PLA relative to the pristine filament likely reflect changes in physical structure resulting from the thermal and shear history experienced during fused-filament fabrication [41,42].

Importantly, no new absorption bands, significant peak shifts, or disappearance of characteristic signals were detected, indicating that no major chemical modifications of the PLA backbone occurred during recycling or electrospinning within the detection limits of FTIR analysis. However, it should be noted that FTIR spectroscopy is inherently limited in its sensitivity to subtle degradation phenomena such as chain scission, reductions in molecular weight, formation of terminal hydroxyl or carboxyl groups, or low-level oxidative degradation. These changes typically require complementary techniques, such as gel permeation chromatography or thermal analysis, for accurate detection [41,43].

Therefore, the FTIR results demonstrate that the recycling and electrospinning processes did not induce detectable chemical structural changes in PLA, while acknowledging that minor molecular-weight reductions or chain-end modifications may still occur without producing measurable spectral alterations.

#### 3.2.2. Differential Scanning Calorimetry (DSC)

**Figure 4:**
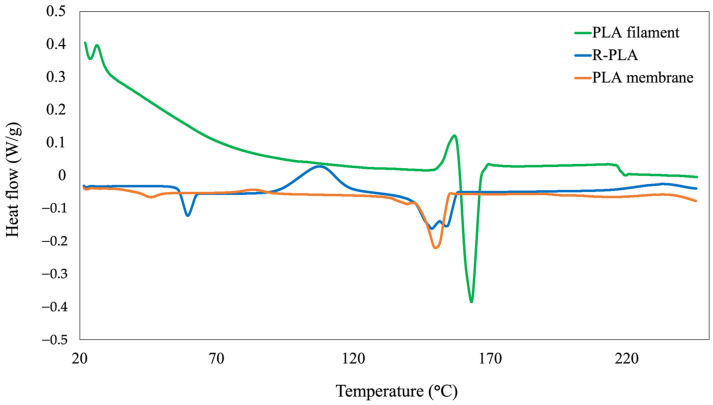
Differential scanning calorimetry (DSC) thermograms of the pristine PLA filament, recycled PLA (R-PLA), and electrospun PLA membrane. The curves show the main thermal transitions, including glass transition temperature (Tg), cold crystallization temperature (Tc), and melting temperature (Tm), illustrating the progressive reduction in crystallinity and thermal stability associated with recycling and electrospinning processes.

Differential scanning calorimetry (DSC) was employed to evaluate the thermal transitions and crystalline structure evolution of the pristine PLA filament, recycled PLA (R-PLA), and electrospun PLA membranes. The corresponding thermograms are shown in Figure 4, and the quantitative thermal parameters extracted from the curves are summarized in Table 2, including glass transition temperature (Tg), cold crystallization temperature (Tc), melting temperature (Tm), melting enthalpy (ΔHm), and degree of crystallinity (χ).

The pristine PLA filament exhibited a well-defined glass transition at 97.9 °C, followed by a distinct cold crystallization peak at 158.8 °C and a sharp melting endotherm centered at 163.2 °C. The relatively high melting enthalpy (40.14 J g^−1^) and crystallinity degree (42.8%) indicate a semi-crystalline structure with significant chain ordering, which is typical of commercial PLA filaments subjected to controlled extrusion and cooling conditions during manufacturing. Similar thermal behavior has been widely reported for injection-molded and extruded PLA products, where processing induces partial chain alignment and promotes crystalline domain formation [38,44].

In contrast, the recycled PLA displayed a pronounced shift in Tg toward lower temperatures (55.9 °C), accompanied by a significant reduction in Tc (108.3 °C) and Tm (148.6 °C). These changes are consistent with thermomechanical degradation occurring during fused filament fabrication (FFF) and subsequent recycling steps. PLA is highly susceptible to hydrolytic and thermal chain scission at processing temperatures above 180 °C, leading to a reduction in molecular weight and increased chain mobility. As a result, shorter polymer chains require lower thermal energy for segmental motion, explaining the observed Tg decrease. Additionally, the lower Tc suggests enhanced recrystallization ability due to increased chain mobility in degraded PLA systems [40,45]. The slight decrease in melting enthalpy to 36.82 J g^−1^ further confirms a partial loss of crystalline order after the printing–recycling cycle.

The electrospun PLA membrane exhibited the most significant thermal changes, with Tg decreasing to 40.6 °C, Tc shifting to 73.7 °C, and Tm broadening and decreasing to 144.0 °C. The melting enthalpy dropped markedly to 27.30 J g^−1^, corresponding to a crystallinity degree of only 29.1%. This progressive reduction in crystallinity reflects the highly amorphous structure produced during electrospinning, where rapid solvent evaporation and strong elongational forces kinetically hinder chain folding and crystal growth. Such behavior is well documented in electrospun PLA fibers, which typically exhibit lower crystallinity than bulk counterparts due to the freezing of polymer chains in non-equilibrium conformations [46,47].

Importantly, the systematic decrease in Tg, Tm, and crystallinity from filament → R-PLA → electrospun membrane provides indirect but strong evidence of molecular weight reduction through chain scission during thermal processing. Although direct molecular weight measurements were not performed, DSC analysis is widely recognized as a reliable indirect indicator of PLA degradation, since reductions in Tg and melting parameters correlate strongly with decreases in polymer chain length and entanglement density [40,45].

Overall, the DSC results quantitatively demonstrate a progressive structural evolution from a relatively ordered semi-crystalline filament to a partially degraded recycled polymer and finally to a predominantly highly amorphous electrospun membrane. This transition is characterized by decreasing thermal transition temperatures, reduced melting enthalpy, and lower crystallinity, which collectively reflect increasing chain disorder and reduced molecular weight. Such structural changes are advantageous for electrospinning, as lower crystallinity and higher chain mobility facilitate fiber formation and contribute to the development of uniform porous networks suitable for filtration applications.

### 3.3. Morphological and Wettability Analysis of Electrospun Membranes

#### 3.3.1. R-PLA5

**Figure 5:**
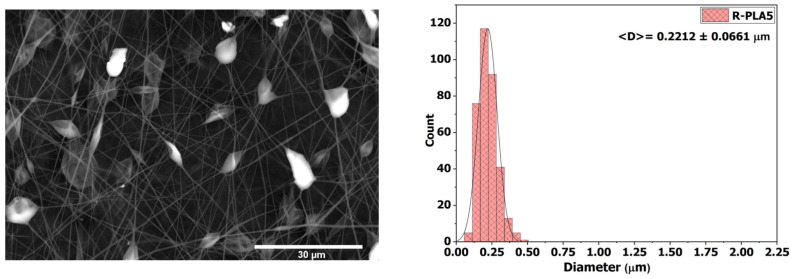
SEM micrograph and average fiber diameter of the R-PLA5 sample.

Figure 5 presents the SEM micrograph and fiber diameter distribution of the membrane obtained from the 5 wt % PLA solution (R-PLA5). Beads are observed across the entire surface, a morphology attributed by Jun et al. to the low viscosity of the solution (41.86 ± 0.11 mPa·s), which compromises jet stability during electrospinning and favors discontinuities in the fibrous network [48]. This morphology was also associated with reduced mechanical strength, as evidenced by the membrane’s poor resistance to handling. The average fiber diameter was 0.221 ± 0.066 µm. The high solution conductivity (7.267 ± 0.057 µS·cm^−1^) contributed to intense jet stretching under the applied electric field, resulting in ultrafine fibers; however, the presence of structural defects further compromised the integrity of the membrane. Wettability tests revealed a contact angle of 112.34° ± 3.49°, confirming the membrane’s hydrophobic character. This behavior is consistent with the nanoscale architecture and the inherent chemical nature of PLA, which reduce the surface free energy and limit interactions with polar liquids.

#### 3.3.2. R-PLA6.25

**Figure 6:**
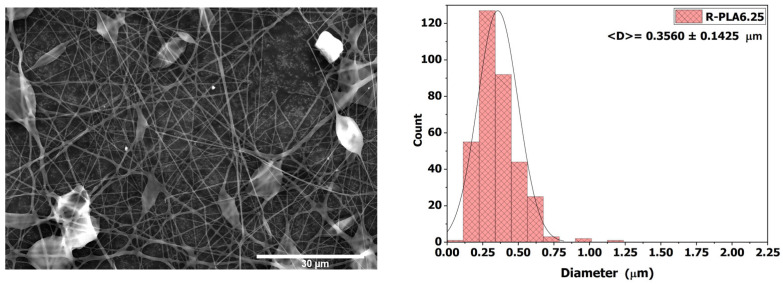
SEM micrograph and average fiber diameter of the R-PLA6.25 sample.

When the polymer solution concentration was increased to 6.25 wt %, fiber formation exhibited greater stability, as shown in Figure 6, where a clear reduction in bead density is observed compared with R-PLA5. This improvement is attributed to the increase in solution viscosity, which reached 62.10 ± 3.75 mPa·s. The higher viscosity promoted fiber thickening, resulting in an average fiber diameter of 0.3560 ± 0.1425 µm. This increase in fiber thickness enhanced the structural quality of the electrospun network and was accompanied by an increase in the contact angle to 119.18° ± 3.65°, a value that exceeds the hydrophobic threshold and confirms the hydrophobic nature of the membrane surface.

#### 3.3.3. R-PLA7.5

**Figure 7:**
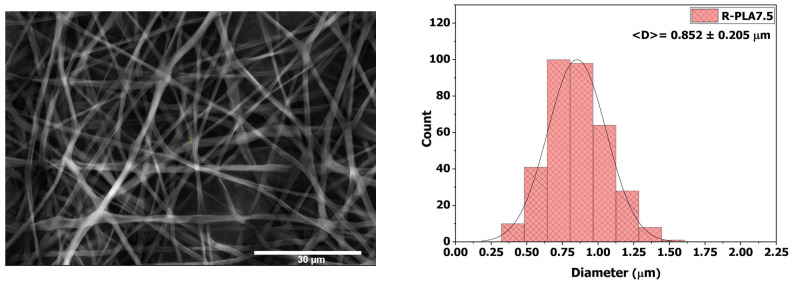
SEM micrograph and average fiber diameter of the R-PLA7.5 sample.

The membrane electrospun from the 7.5 wt % PLA solution (R-PLA7.5) exhibited a uniform fiber network without bead formation (Figure 7). The increase in viscosity to 125.67 ± 1.26 mPa·s contributed to a more stable electrospinning process, enabling the formation of a well-defined and consistent Taylor cone. Consequently, the average fiber diameter increased to 0.852 ± 0.205 µm, in agreement with the reduced conductivity of the solution (1.9 µS·cm^−1^). The enhanced morphological homogeneity and absence of structural defects resulted in enhanced surface properties, with the contact angle reaching 122.88° ± 1.43°, confirming a pronounced hydrophobic character.

The progressive enhancement of hydrophobicity from R-PLA5 to R-PLA7.5 highlights the critical role of fiber uniformity and interfacial stability. While R-PLA5 exhibited rapid structural collapse during contact-angle measurement—indicative of weak fiber cohesion and insufficient interchain entanglement—R-PLA7.5 maintained its fibrous architecture throughout the test, demonstrating improved resistance to wetting and confirming its highly hydrophobic character.

#### 3.3.4. R-PLA8.75

**Figure 8:**
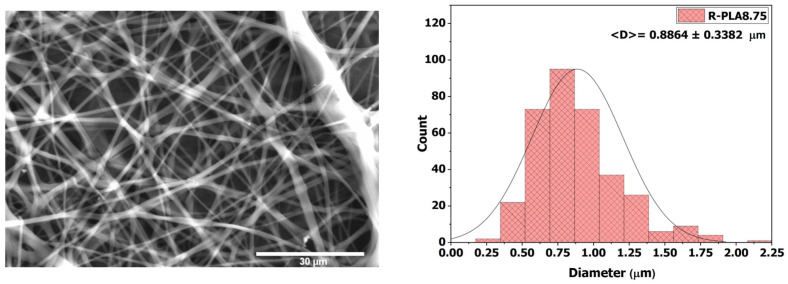
SEM micrograph and average fiber diameter of the R-PLA8.75 sample.

When the polymer solution concentration was increased to 8.75 wt %, fiber formation exhibited even greater stability, as evidenced by a more uniform morphology and an almost complete absence of beads, as shown in Figure 8. This substantial improvement is associated with the marked increase in viscosity, which reached 211 ± 2.89 mPa·s. The higher viscosity promoted the formation of more controlled and homogeneous fibers, with an average diameter of 0.8864 ± 0.3382 µm. Although this value is comparable to that obtained at the previous concentration, it is accompanied by a more well-defined and continuous structure, which contributes positively to the overall performance of the electrospun material.

On the other hand, the contact angle increased slightly to 123.45 ± 1.20°, suggesting that the reduction in porosity—which contributes to air trapping at the surface and enhances apparent hydrophobicity through air entrapment—reaches a limiting threshold. Beyond this point, further decreases in porosity no longer lead to a significant increase in the contact angle, indicating that the surface has effectively reached its maximum hydrophobic capacity.

#### 3.3.5. R-PLA10

**Figure 9:**
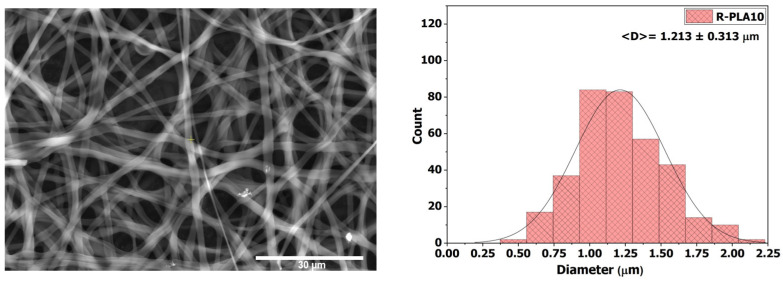
SEM micrograph and average fiber diameter of the R-PLA10 sample.

The membrane electrospun from the 10 wt % PLA solution (R-PLA10) exhibited a dense and uniform fibrous network without bead formation, as shown in Figure 9. This behavior is primarily attributed to the marked increase in solution viscosity (399.83 ± 4.04 mPa·s), which enhanced the stability of the electrospinning jet and enabled continuous fiber formation without structural discontinuities. The average fiber diameter reached 1.213 ± 0.313 µm, a result that correlates with the lower conductivity of the solution (1.667 ± 0.058 µS·cm^−1^), which limited electrostatic stretching during fiber formation. This morphology reflects a well-developed nanofibrous architecture with increased fiber thickness, a feature commonly associated with enhanced structural cohesion and improved morphological stability of electrospun mats.

The contact angle measured for R-PLA10 was 124.38° ± 2.01°, confirming a strongly hydrophobic surface and revealing a slight enhancement in water repellency compared to R-PLA7.5. This increase can be attributed to the larger fiber diameter and the enhanced surface roughness of the membrane. Additionally, the observed increase in apparent contact angle may be tentatively associated with a Cassie–Baxter wetting regime, in which air pockets trapped within the porous fibrous network reduce the effective solid–liquid contact area, leading to enhanced water repellency. However, it should be noted that no direct measurements of surface roughness, sliding angle, or dynamic contact angle hysteresis were performed in this study. Therefore, the Cassie–Baxter interpretation should be regarded as a plausible hypothesis rather than a conclusively demonstrated wetting mechanism [49].

These findings underscore the significant influence of solution parameters—particularly viscosity and conductivity—on the morphological and interfacial characteristics of electrospun PLA membranes. An increase in polymer concentration promotes the formation of bead-free, structurally coherent, and highly hydrophobic mats, which present promising attributes for applications requiring moisture resistance.

Although all membranes exhibited contact angles significantly above 90°, indicating clear hydrophobicity, the measured values remained below the threshold typically required to classify a surface as superhydrophobic. Therefore, the observed wetting behavior is more accurately described as highly hydrophobic rather than superhydrophobic.

### 3.4. Correlation Analysis

The correlation analysis of parameters derived from the characterization of both polymer solutions and the resulting electrospun membranes was performed using Equation (1). The evaluated dataset included five PLA concentrations (n = 5), and the corresponding physicochemical parameters are summarized in Table 3. The analysis comprised solution viscosity and conductivity, together with membrane contact angle and average fiber diameter.

**Figure 10:**
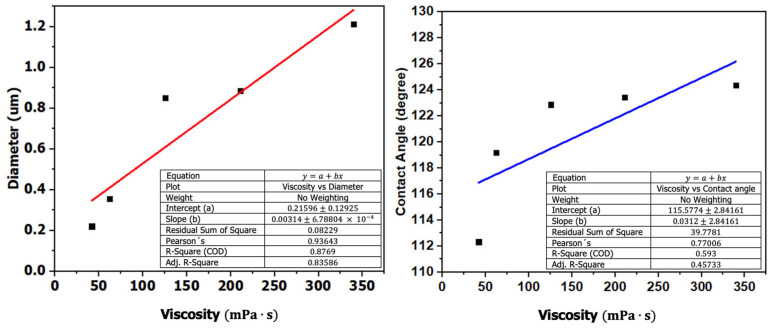
Relationship between viscosity, contact angle, and fiber diameter.

Pearson correlation coefficients (r), associated *p*-values, coefficients of determination (R^2^), and degrees of freedom (df = 3) were calculated. A positive correlation was observed between viscosity and contact angle (r = 0.7701, *p* = 0.1277, R^2^ = 0.5930), indicating a moderate linear trend that did not reach statistical significance at the 95% confidence level. In contrast, viscosity exhibited a strong positive correlation with average fiber diameter (r = 0.9364, *p* = 0.0191, R^2^ = 0.8769), suggesting a statistically significant association between these variables. The high R^2^ value obtained for fiber diameter reflects a strong linear relationship with solution viscosity, indicating that variations in rheological behavior are closely associated with changes in fiber morphology. However, given the limited sample size (n = 5), these results should be interpreted as indicative trends rather than definitive predictive relationships. Figure 10 illustrates the relationships between viscosity, contact angle, and fiber diameter.

**Figure 11:**
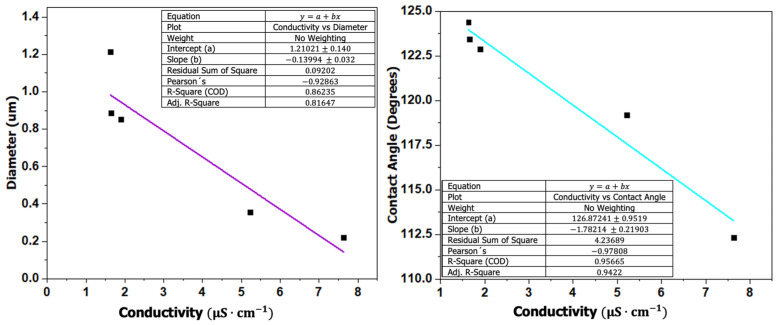
Relationship between conductivity, contact angle, and fiber diameter.

Furthermore, solution conductivity exhibited a strong negative correlation with contact angle (r = −0.9781, *p* = 0.00388, R^2^ = 0.9566, df = 3), indicating a statistically significant inverse relationship. This result suggests that lower conductivity values are associated with higher apparent contact angles. A similar negative correlation was observed between conductivity and fiber diameter (r = −0.9286, *p* = 0.0226, R^2^ = 0.8624, df = 3), also statistically significant at the 95% confidence level. These findings indicate that decreasing conductivity is associated with an increase in fiber diameter, which may be related to reduced charge density within the electrospinning jet and consequently lower electrostatic stretching forces during fiber formation. Figure 11 presents the relationships between conductivity, contact angle, and fiber diameter.

**Figure 12:**
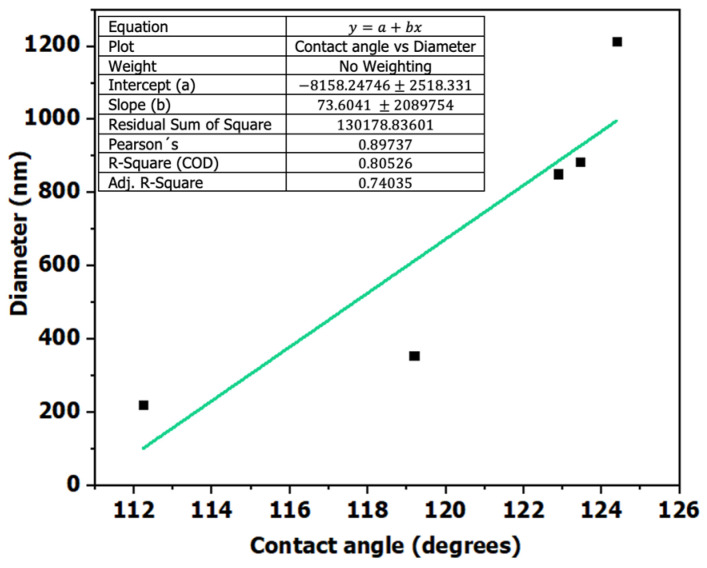
Analysis of the relationship between contact angle and fiber diameter.

Finally, the relationship between contact angle and fiber diameter was evaluated (Figure 12), revealing a strong positive correlation (r = 0.8974, *p* = 0.0389, R^2^ = 0.8053, df = 3). This statistically significant association suggests that larger fiber diameters are linked to higher apparent contact angles. This behavior may be related to morphological modifications that influence surface roughness and pore distribution, potentially promoting air entrapment within the fibrous network. Nevertheless, this interpretation should be considered a plausible hypothesis rather than a confirmed wetting mechanism, as no direct surface roughness measurements or dynamic wetting analyses were conducted.

Overall, although the limited number of formulations restricts the statistical power of the correlation analysis, the consistency and magnitude of the observed coefficients support the existence of systematic associations between solution properties and membrane morphology.

### 3.5. Effect of Layer Number on Membrane Morphology

**Figure 13:**
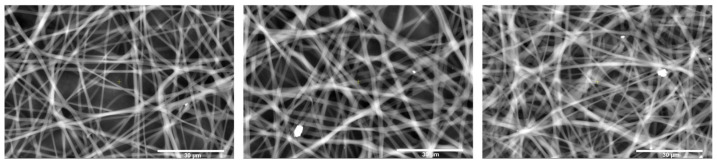
SEM images of electrospun membranes with one, two, and three layers.

In an extension of the correlation analysis, membrane porosity was systematically adjusted by varying the number of electrospun layers in the R-PLA10 sample, which had previously shown the most favorable performance in preliminary evaluations. Using this concentration as a reference, membranes were fabricated with one, two, and three electrospun layers. To increase the number of layers under the previously established electrospinning parameters, the first layer was allowed to dry for 20 min before initiating the deposition of the second layer. The same procedure was followed for the addition of the third layer. This strategy aimed to increase fiber packing density within the membrane, thereby reducing pore size and enhancing pore density at the surface. These structural modifications are fundamental for improving the homogeneity of the porous network and influencing key functional properties such as filtration efficiency and wetting behavior. The morphological changes associated with the increasing number of layers are illustrated in Figure 13, which shows SEM micrographs of the electrospun membranes with one, two, and three layers. These images clearly reveal the progressive densification of the fibrous matrix, highlighting the role of layer stacking in tuning membrane architecture. Accordingly, controlling the number of electrospun layers proves to be a decisive parameter for designing membranes with application-specific performance.

**Figure 14:**
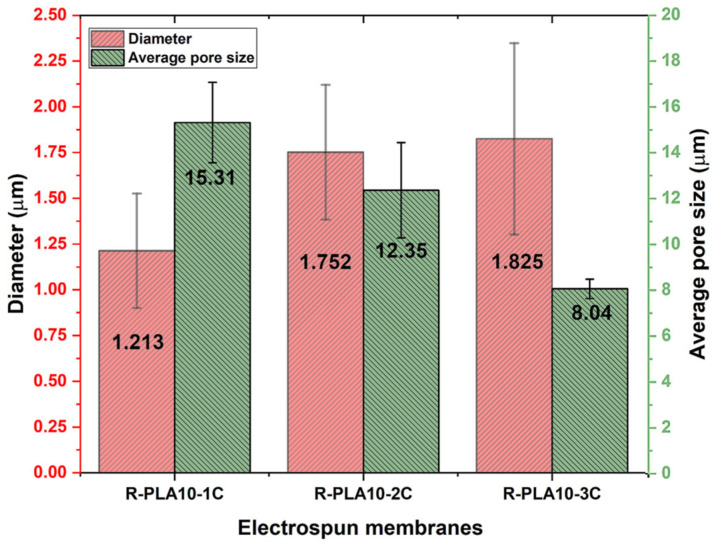
Comparison of fiber diameters and pore sizes in R-PLA10 membranes with different numbers of layers.

Porosity analysis of the electrospun membranes with different numbers of layers was performed using scanning electron microscopy (SEM) equipped with a concentric backscattered electron (CBS) detector. This detector was selected for its ability to provide high-resolution surface imaging, thereby facilitating accurate assessment of membrane porosity. SEM images were acquired at 400× magnification to capture a broader and more representative surface area. To ensure the robustness of the analysis, five measurements were conducted at different regions of each electrospun membrane. The corresponding results are presented in Figure 14.

**Figure 15:**
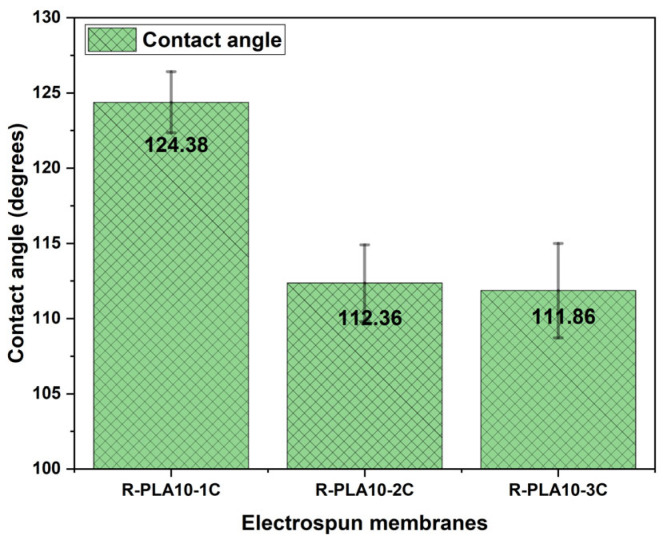
Contact angle of electrospun R-PLA membranes with different numbers of layers.

As illustrated in Figure 15, increasing the number of electrospun layers leads to a clear reduction in pore size, while the average fiber diameter progressively increases. This behavior is associated with the accumulation of residual electrostatic charge on the previously deposited layers and the consequent delay in charge dissipation within the fibrous mat. When charge relaxation is slow, the deposited layers partially shield the electric field in the deposition region, thereby reducing the effective electrostatic stress acting on the incoming polymer jet. Under these conditions, the extensional deformation rate decreases, and macromolecular viscoelastic relaxation becomes increasingly dominant, promoting jet contraction (entropic recoil) and resulting in the formation of thicker fibers. This interpretation is consistent with electrospinning models that emphasize the importance of charge relaxation times relative to the hydrodynamic and viscoelastic relaxation times of the jet, as well as with theoretical and experimental analyses showing that rheological effects can limit jet stretching and lead to increased fiber diameters [50,51].

In addition, a gradual decrease in the contact angle was observed with increasing layer number (Figure 15), which can be attributed to the evolution of surface topography and pore–fiber architecture. As the multilayer structure becomes denser and exhibits greater heterogeneity in fiber diameter, the effective surface roughness and wetting regime are altered, resulting in changes in the apparent wettability of the membranes.

### 3.6. Particle Filtration Efficiency (PFE) Performance

The particle filtration efficiency (PFE) of electrospun R-PLA10 membranes with one, two, and three layers was evaluated using the custom-built filtration system described in Section 2.7. Tests were conducted using a 2% NaCl polydisperse aerosol at a constant airflow rate of 85 L min^−1^, corresponding to a frontal air velocity of 1 cm s^−1^, and an effective filtration area of 50 cm^2^. Under these fixed operating conditions, particle concentrations upstream and downstream of the membrane were selectively monitored for three predefined particle size fractions: PM1, PM2.5, and PM5.

According to the calibration data of the particle counter, these channels correspond to mean aerodynamic diameters of 1.0, 2.5, and 5.0 µm, with associated standard deviations of ±0.01, ±0.025, and ±0.050 µm, respectively.

**Figure 16:**
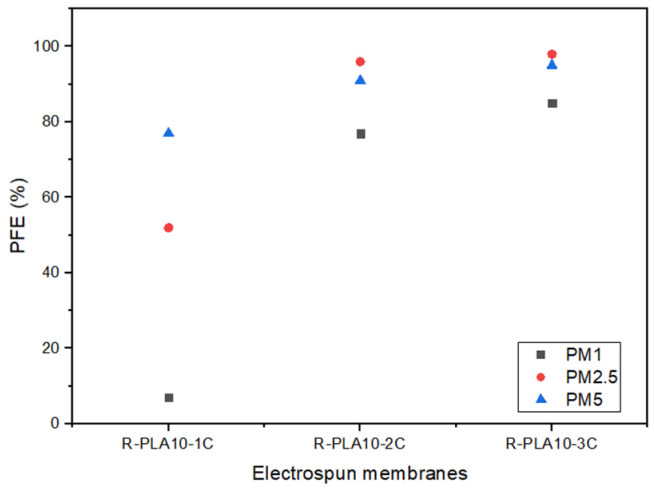
Particle filtration efficiency (PFE) of electrospun R-PLA10 membranes with one (1C), two (2C), and three (3C) layers for PM1, PM2.5, and PM5 particle sizes.

All filtration efficiency values reported in Figure 16 correspond to the average of three independent measurements. The associated standard deviations were below 1% in all cases, confirming good repeatability of the experimental setup. Specifically, for PM1 particles, the standard deviations were 0.07%, 0.70%, and 0.85% for the one-, two-, and three-layer membranes, respectively. For PM2.5, the corresponding deviations were 0.16%, 0.29%, and 0.29%, while for PM5 particles values, of 0.23%, 0.27%, and 0.29% were obtained. These low dispersion values indicate stable aerosol generation and consistent particle counting during the filtration tests. It should be noted that the filtration experiments were conducted under constant airflow and geometric conditions to ensure internal comparability among membrane configurations. However, since the setup does not strictly follow standardized ASTM or EN testing protocols, the reported PFE values should be interpreted as comparative performance indicators rather than directly equivalent to certified filtration ratings.

As shown in Figure 16, PFE increased consistently with the number of electrospun layers. For PM5 and PM2.5, membranes composed of two and three layers achieved filtration efficiencies exceeding 90%, whereas the single-layer configuration exhibited markedly lower retention. The capture of PM1 particles proved more demanding, with the single-layer membrane showing limited efficiency; however, the addition of a second and third layer resulted in a pronounced improvement, highlighting the strong influence of membrane thickness on the removal of fine particles.

It should be emphasized that particle capture in electrospun membranes is not governed exclusively by pore-size–based sieving mechanisms. As previously shown in Figure 14, the mean pore size of the membranes ranges between approximately 8 and 15 µm, which is significantly larger than the tested particle diameters.

The high retention observed for micrometric PM5 particles is primarily attributed to inertial impaction and direct interception mechanisms, which dominate the capture of particles in the micrometer range as they deviate from airflow streamlines and collide with fibers [52,53]. In contrast, the relatively lower efficiency observed for PM1 particles in the single-layer membrane is mainly associated with their smaller inertia and higher mobility, which reduce the probability of collision with fibers during short residence times within the porous network.

Increasing the number of electrospun layers effectively enhances membrane thickness, fiber packing density, and tortuosity, thereby strengthening interception and impaction mechanisms while also increasing the probability of diffusional capture for smaller particles. Although electrostatic interactions may contribute to particle adhesion, particularly in electrospun materials with high surface area, their role in the present system is considered secondary compared to classical mechanical filtration mechanisms.

These effects are directly associated with the progressive increase in structural complexity and reduction in effective flow pathways as the number of electrospun layers increases, as discussed in Section 3.5. The random and continuous deposition of fibers generates a denser and more tortuous network, promoting particle capture while maintaining structural coherence of the membrane under aerosol flow, thereby preserving filtration performance.

### 3.7. Quality Factor (QF)

The pressure drop values obtained during the PFE tests (Table 4) reflect the well-established trade-off between filtration efficiency and airflow resistance in fibrous filtering media. As the structural density of the membrane increases, which is commonly associated with a reduction in effective porosity, particle retention tends to improve; however, this enhancement is accompanied by an increase in pressure drop, which directly affects air permeability and, consequently, the operational performance of filtration systems.

Using the pressure drop values listed in Table 4 together with the corresponding filtration efficiencies, the quality factor (QF) was calculated to provide an integrated assessment of filtration performance, simultaneously accounting for particle capture capability and airflow resistance. The resulting QF values for the different particle size fractions are summarized in Table 5.

The calculated quality factor (QF) values reveal a non-linear dependence on both the number of electrospun layers and the particle size fraction (PM1, PM2.5, and PM5). For R-PLA10-1C, the QF values are consistently the lowest across all fractions, reaching 0.032 Pa^−1^ (PM1), 0.033 Pa^−1^ (PM2.5), and 0.065 Pa^−1^ (PM5). This behavior is indicative of a relatively open fibrous network that provides low airflow resistance but limited particle capture efficiency, particularly for fine aerosols, thereby restricting overall filtration performance when efficiency and pressure drop are considered simultaneously.

Increasing the deposition to two electrospun layers (R-PLA10-2C) leads to a marked improvement in QF for all particle size ranges, with values of 0.044 Pa^−1^ (PM1), 0.097 Pa^−1^ (PM2.5), and 0.072 Pa^−1^ (PM5). This enhancement suggests an optimal balance between increased filtration efficiency and a moderate rise in pressure drop, likely associated with a more effective reduction in inter-fiber voids and improved particle interception mechanisms while maintaining adequate airflow pathways.

In contrast, the three-layer membrane (R-PLA10-3C) does not preserve this improvement, as QF values decrease to 0.023 Pa^−1^ (PM1), 0.047 Pa^−1^ (PM2.5), and 0.036 Pa^−1^ (PM5). This trend indicates an over-compaction of the fibrous structure, where the increase in pressure drop outweighs the marginal gains in particle capture, ultimately diminishing the efficiency–permeability trade-off across all evaluated particle fractions.

Overall, these results demonstrate that membrane performance is strongly governed by the number of deposited electrospun layers, with the intermediate configuration of two layers (R-PLA10-2C) providing the most favorable balance between particle retention and air permeability over the analyzed particle size ranges.

## 4. Conclusions

Recycled PLA derived from 3D-printing waste was successfully converted into functional electrospun membranes while preserving its chemical structure. Thermal analysis revealed reduced crystallinity after electrospinning, consistent with increased molecular disorder associated with fiber formation.

PLA concentration in the electrospinning solution governed fiber morphology and surface properties. Higher concentrations promoted bead-free fibers with larger diameters and increased hydrophobicity. Exploratory correlation analysis indicated consistent associations between solution viscosity, fiber diameter, and contact angle.

Membrane architecture was further controlled by the number of electrospun layers. Increasing layer deposition generated denser fibrous networks with reduced pore size, directly influencing particle retention mechanisms dominated by interception, inertial impact, and diffusion.

Under controlled airflow conditions (85 L min^−1^, 1 cm s^−1^ frontal velocity, 50 cm^2^ filtration area), multilayer R-PLA10 membranes exhibited high filtration efficiencies for PM2.5 and PM5 particles, while PM1 capture improved with increasing thickness. As expected, enhanced retention was accompanied by increased airflow resistance. Quality factor analysis indicated that intermediate multilayer configurations provided a favorable balance between efficiency and permeability.

Overall, these results demonstrate that electrospinning enables the transformation of recycled PLA into structurally tunable membranes with adjustable morphological and filtration properties, offering a sustainable pathway for the valorization of additive manufacturing waste.

## Figures and Tables

**Table 1:** Designation of electrospun samples according to PLA concentration.

Sample ID	PLA Concentration (%wt)
R-PLA5	5.00
R-PLA6.25	6.25
R-PLA7.5	7.50
R-PLA8.75	8.75
R-PLA10	10.00

**Table 2:** Thermal properties of pristine PLA filament, recycled PLA (R-PLA), and electrospun PLA membrane determined from DSC analysis, including glass transition temperature (Tg), cold crystallization temperature (Tc), melting temperature (Tm), melting enthalpy (ΔHm), and degree of crystallinity (χ).

Material	Tg (°C)	Tc (°C)	Tm (°C)	ΔHm (J/g)	χ (%)
PLA filament	97.91	158.78	163.19	40.14	42.84
R-PLA	55.95	108.32	148.63	36.82	39.30
PLA membrane	40.62	73.70	143.97	27.30	29.14

**Table 3:** Experimental parameters used for Pearson correlation analysis, including solution properties and membrane characteristics.

Sample	Viscosity (mPa·s)	Conductivity (µS·cm^−1^)	Contact Angle (Degree)	Φ (μm)
R-PLA5	41.87	7.63	112.34	0.221
R-PLA6.25	62.16	2.60	119.18	0.356
R-PLA7.5	125.67	1.90	122.88	0.852
R-PLA8.75	210.66	1.65	123.45	0.886
R-PLA10	339.83	1.63	124.38	1.213

**Table 4:** Pressure drop (ΔP) of electrospun R-PLA10 membranes measured during particle filtration efficiency tests.

Sample	Pressure Drop, ΔP (Pa)
R-PLA10-1C	22.555 ± 2.942
R-PLA10-2C	33.342 ± 3.923
R-PLA10-3C	82.873 ± 7.845

**Table 5:** Quality factor (QF) values of electrospun R-PLA10 membranes for different particle size fractions.

Sample	QF-PM1 (Pa^−1^)	QF-PM2.5 (Pa^−1^)	QF-PM5 (Pa^−1^)
R-PLA10-1C	0.032	0.033	0.065
R-PLA10-2C	0.044	0.097	0.072
R-PLA10-3C	0.023	0.047	0.036

## Data Availability

The data presented in this study are available within this article.
